# Adenosine A1 Receptor Antagonism Abolished the Anti-seizure Effects of Exogenous Ketone Supplementation in Wistar Albino Glaxo Rijswijk Rats

**DOI:** 10.3389/fnmol.2017.00235

**Published:** 2017-07-25

**Authors:** Zsolt Kovács, Dominic P. D’Agostino, Arpád Dobolyi, Csilla Ari

**Affiliations:** ^1^Savaria Department of Biology, Eötvös Loránd University Budapest, Hungary; ^2^Hyperbaric Biomedical Research Laboratory, Department of Molecular Pharmacology and Physiology, Morsani College of Medicine, University of South Florida, Tampa FL, United States; ^3^Laboratory of Neuromorphology and Human Brain Tissue Bank, Department of Anatomy, Histology and Embryology, Semmelweis University Budapest, Hungary; ^4^Laboratory of Molecular and Systems Neurobiology, Department of Physiology and Neurobiology, Hungarian Academy of Sciences, Eötvös Loránd University Budapest, Hungary; ^5^Department of Psychology, University of South Florida, Tampa FL, United States

**Keywords:** ketone supplements, epilepsy, adenosine, WAG/Rij rats, ketosis, seizure

## Abstract

The state of therapeutic ketosis can be achieved by using the ketogenic diet (KD) or exogenous ketone supplementation. It was suggested previously that the adenosinergic system may be involved in the mediating effect of KD on suppressing seizure activity in different types of epilepsies, likely by means of adenosine A_1_ receptors (A_1_Rs). Thus, we tested in the present study whether exogenous ketone supplements (ketone ester: KE, 2.5 g/kg/day; ketone salt/KS + medium chain triglyceride/MCT: KSMCT, 2.5 g/kg/day) applied sub-chronically (for 7 days) by intragastric gavage can modulate absence epileptic activity in genetically absence epileptic Wistar Albino Glaxo/Rijswijk (WAG/Rij) rats. The number of spike-wave discharges (SWDs) significantly and similarly decreased after both KE and KSMCT treatment between 3rd and 7th days of gavage. Moreover, blood beta-hydroxybutyrate (βHB) levels were significantly increased alike after KE and KSMCT gavage, compared to control levels. The SWD number and βHB levels returned to the baseline levels on the first day without ketone supplementation. To determine whether A_1_Rs can modify ketone supplement-evoked changes in absence epileptic activity, we applied a non-pro-epileptic dose of a specific A_1_R antagonist DPCPX (1,3-dipropyl-8-cyclopentylxanthine) (intraperitoneal/i.p. 0.2 mg/kg) in combination with KSMCT (2.5 g/kg/day, gavage). As expected, DPCPX abolished the KSMCT-evoked decrease in SWD number. Thus, we concluded that application of exogenous ketone supplements may decrease absence epileptic activity in WAG/Rij rats. Moreover, our results suggest that among others the adenosinergic system, likely *via* A_1_Rs, may modulate the exogenous ketone supplements-evoked anti-seizure effects.

## Introduction

Ketogenic diet (high-fat, adequate protein, and low carbohydrate diet) and ketone supplementation (exogenous ketone supplement and/or MCT) may evoke nutritional ketosis (Kwiterovich et al., 2003; Poff et al., 2015; Kesl et al., 2016), which enhances conversion of ketone bodies such as beta-hydroxybutyrate and acetoacetate (AcAc) into acetyl-CoA to support ATP production (Yudkoff et al., 2007). Under this condition, the cells utilize ketone bodies as an alternative fuel to enhance brain energy metabolism. The classical KD and variants have proven beneficial in treating epilepsy and other disorders by decreasing seizure activity (Lefevre and Aronson, 2000; Kossoff et al., 2011), likely by modulation of GABAergic, glutamatergic, and adenosinergic processes (Yudkoff et al., 2007; Masino et al., 2011; Rho, 2017). The exact mechanism(s) of the KD/nutritional ketosis on epilepsy are largely unknown and remain an area of intense investigation and interest within the pharmaceutical industry.

Genetically absence epileptic WAG/Rij rats spontaneously generate absence-like seizures, and manifest spike-wave asymmetric discharges (SWDs) on the EEG. A typical SWD contains a train of asymmetric spikes and slow waves starting and ending with sharp spikes (frequency: 7–11 Hz and duration 1–30 s in WAG/Rij rats) (Coenen and Van Luijtelaar, 2003). It was demonstrated that the hyperexcitable neurons of cortical focus in the somatosensory cortex initiate SWDs (cortical focus theory) (Meeren et al., 2002).

It has been demonstrated, that oral administration of βHB and AcAc in their free acid form is difficult to produce sustained ketosis, in contrast to ketone ester and ketone salt supplementation (D’Agostino et al., 2013; Kesl et al., 2016). It is well-known that adenosinergic system may be involved not only in the alleviating effect of KD/ketosis on epileptic seizures (mainly by means of A_1_Rs), but also in the modulation of absence epilepsy mechanisms (D’Alimonte et al., 2009; Masino et al., 2011; Kovács et al., 2014; Kim et al., 2016). Thus, we addressed in the present study whether (i) sub-chronically (for 7 days) applied exogenous ketone supplements (KE; KS + MCT: KSMCT) by intragastric gavage can modulate absence epileptic activity and (ii) inhibition of A_1_Rs by its specific antagonist can evoke changes in seizure frequency in WAG/Rij rats. At first, to investigate the influence of the ketone supplements on absence epileptic activity, we administered KE (2.5 g/kg/day) and KSMCT (2.5 g/kg/day) alone by gavage. We measured not only glucose, but also βHB levels in the blood collected from tail veins to establish the ketonemia-inducing effect of ketone supplementation as ketonemia is the clinical hallmark associated with metabolic management of seizures (Freeman et al., 1994). As we have no data on the putative effects of intraperitoneally (i.p.) injected specific A_1_R antagonist DPCPX on absence epileptic activity in WAG/Rij rats, we also investigated the effect of two doses (i.p. 0.2 and 0.5 mg/kg alone) on SWD number. Subsequently, to decide whether A_1_Rs can modify ketone supplements-evoked changes in SWD number, we applied a lower dose (i.p. 0.2 mg/kg) of DPCPX in combination with KSMCT (2.5 g/kg/day, gavage). We hypothesized that (i) exogenous ketone supplementation evokes ketosis, and decreases absence epileptic seizures, and (ii) A_1_R blockade modifies the influence of exogenous ketone supplementation on SWD number. Indeed, we demonstrated that exogenous ketone supplements increased blood βHB levels and decreased SWD number when given sub-chronically and inhibition of A_1_Rs abolished the exogenous ketone supplements-evoked changes in SWD number in WAG/Rij rats.

## Materials and Methods

### Animals

All animal treatments and surgery procedures were carried out according to the local ethical rules, which are in conformity with the guidelines of the Hungarian Act of Animal Care and Experimentation (1998, XXVIII, section 243), European Communities Council Directive 24 November 1986 (86/609/EEC) and EU Directive 2010/63/EU on the use and treatment of animals in experimental laboratories. The experimental design was approved by the Animal Care and Experimentation Committee of the Eötvös Loránd University (Savaria Campus) and National Scientific Ethical Committee on Animal Experimentation (Hungary) under license number VA/ÉBNTF02/85-8/2016, and was compliant with the Ethics Codex of institution. All efforts were made to minimize pain and suffering and to reduce the number of animals used.

Wistar Albino Glaxo/Rijswijk male rats (*n* = 33; 10 months old, 315–350 g; breeding colony of WAG/Rij rats at Eötvös Loránd University, Savaria Campus, Szombathely, Hungary) were housed in groups 3–4 and they were single housed after surgery. Standard laboratory conditions were as follows: 12:12 h light-dark cycle (light was on from 08.00 AM to 08.00 PM); free access to water and food; air-conditioned room (at 22 ± 2°C).

### Electrode Implantation and EEG Recording

**FIGURE 1:**
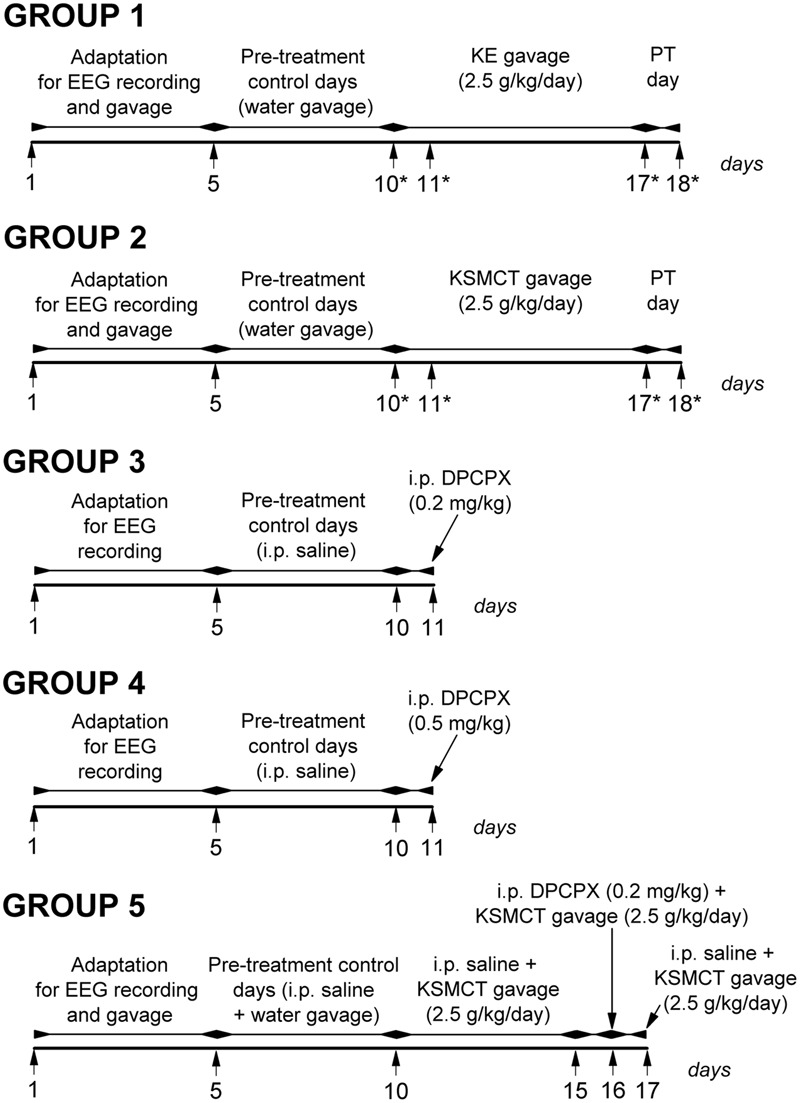
Design of experiments on five animal groups. ^∗^, days of blood glucose and βHB level measuring; DPCPX, 1,3-dipropyl-8-cyclopentylxanthine; KE, ketone ester (1,3-butanediol – acetoacetate diester); KSMCT, ketone salt/KS + medium chain triglyceride/MCT); PT day, post-treatment control experiment/day.

Electrode implantation was carried out under Isoflurane-air mixture (2.0–2.5%) anesthesia with stainless steel screw electrodes for EEG recording (Kovács et al., 2006). Briefly, screw electrodes were placed into the bone above primary motor cortex and somatosensory cortex (A 0.8, L 1.8 and A 0.2, L 6.2, respectively) (Paxinos and Watson, 1999). A stainless steel plate (3 mm × 4 mm with one side insulated) and a screw electrode were implanted under the skin and over the masseter muscle as well as above the cerebellar cortex as reference electrode and ground electrode, respectively. The plate and electrodes were soldered to a 10-pin socket. Dentacrylate cement (Ivoclar, Liechtenstein) was used to fix electrodes and attach the socket to the skull. Lidocaine ointment (5%; EGIS, Hungary) was applied as post-operative pain relief. Rats were allowed to recover for 2 weeks. For the adaptation of rats to the experimental procedures animals were handled daily and were connected to an electroencephalograph for 5 days (adaptation to EEG recording) (**Figure 1**).

**FIGURE 2:**
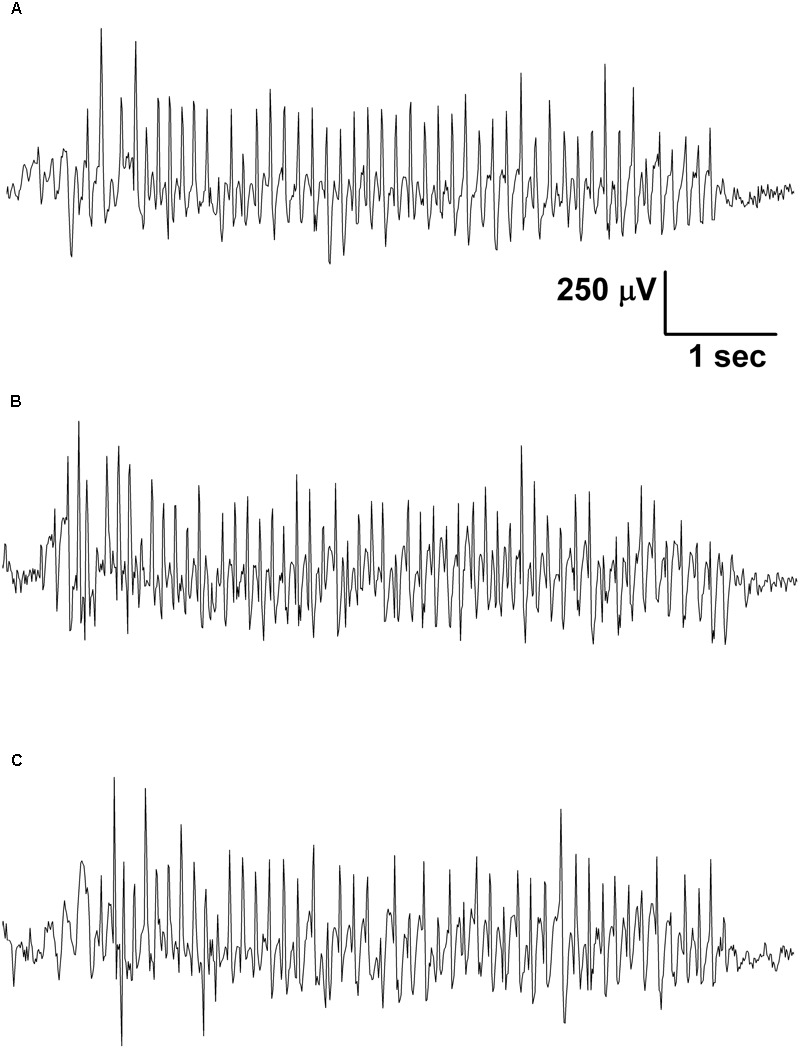
Typical SWDs were observed in all animals 30 min after the connection of rats to the electroencephalograph in the control period (**A**; group 1) as well as after gavage of KE (**B**; 1,3-butanediol – acetoacetate diester, 2.5 g/kg/day, group 1) and KSMCT (**C**; ketone salt + medium chain triglyceride, 2.5 g/kg/day, group 2).

Electroencephalograms were recorded by an electro encephalograph (NIHON-KOHDEN, Japan) attached to a CED 1401 mkII (Cambridge Electronic Design, Ltd, United Kingdom) data capture and analysis device (the bandwidth of the EEG recording: 0.3–150 Hz; the sampling rate 500 Hz) (Kovács et al., 2015). We recorded EEG between 2.30 PM and 5.00 PM. As handling may evoke stress-induced changes in behavior for about 30 min, which can modify SWD number (Coenen and Van Luijtelaar, 2003; Kovács et al., 2015), evaluation of SWD number, average time and total time of SWDs and sleep-waking stages were carried out between 30 and 150 min. These periods were split into 60 min sections and were evaluated separately (Kovács et al., 2006). However, normal grooming and behavior and typical SWDs (**Figure 2**) were observed in all animals 30 min after the connection of rats to the electroencephalograph (e.g., after KE or KSMCT gavage). SWDs were separated from the EEG manually and checked by FFT analysis.

### Measuring of Blood Glucose and βHB Levels

Blood glucose (mg/dl) and βHB (mmol/l) levels were measured from blood taken from the tail vein with a commercially available glucose and ketone monitoring system (Precision Xtra^TM^, Abbott Laboratories, Abbott Park, IL, United States) (Ari et al., 2016). This ketone monitoring system only measures blood levels of βHB, thus, total blood ketone levels (βHB + AcAc + acetone) would be higher than we measured.

### Experimental Design

Both KE (1,3-butanediol – acetoacetate diester) and KS (Na^+^/K^+^ – βHB mineral salt) were developed by D’Agostino et al. (2013; University of South Florida/USF, United States) in collaboration with Savind, Inc. (Urbana, IL, United States). Ketone salt was mixed into a 50% solution (375 mg/g pure βHB and 125 mg/g of Na^+^/K^+^ in a 1:1 ratio). MCT oil (pharmaceutical grade; approximately 60% caprylic triglyceride and 40% capric triglyceride) was purchased from Now Foods (Bloomingdale, IL, United States). KS and MCT were also mixed in a 1:1 ratio (KSMCT) at the USF (United States). At first, we tested the tolerability and effectiveness of exogenous ketone supplementation (*ad libitum* access to normal rat chow + KE and KSMCT given by intragastric gavage once/day) for 7 days in the first phase of the study to assess ketonemia. Therefore, in the second phase of the study, KE (2.5 g/kg/day) and KSMCT (2.5 g/kg/day) were applied as ketone supplements alone by gavage followed by EEG recording.

Rats were assigned into five groups (**Figure 1**). To adapt the animals to gavage method (animal group 1/group 1, group 2 and group 5) we applied water intragastric gavage for 5 days before control treatments (between 1st and 5th day of the experiment). Then, to establish averaged control SWD numbers, SWD durations and sleep-waking stages, rats were (i) ‘treated’ by gavage of water (2.5 g/kg/day) (group 1, *n* = 7 and group 2, *n* = 7), (ii) injected i.p. by 0.5 ml saline/100 g body weight (b. w.; group 3, *n* = 6 and group 4, *n* = 6) or (iii) i.p. injected by 0.5 ml saline/100 g b. w., which injection was followed by the water gavage (2.5 g/kg/day) (30 min later; group 5, *n* = 7) on 5 consecutive days (5-day control period, pre-treatment control days; between 6th and 10th day of the experiment) (**Figure 1**). After control periods, animals of group 1 and group 2 were treated with KE (2.5 g/kg/day, group 1) and KSMCT (2.5 g/kg/day, group 2) alone for 7 consecutive days (between 11th and 17th day of the experiment) (**Figure 1**). Finally, the 7 days treatments by both KE and KSMCT were followed by a day of water gavage (2.5 g/kg/day; post-treatment control experiment/day: PT day) to investigate putative sustained effect of ketone supplementation on SWD number. In relation to group 3 and group 4, to investigate the effect of the specific A_1_R antagonist DPCPX on SWD number, 0.2 mg/kg (group 3) and 0.5 mg/kg (group 4) of DPCPX in 0.5 ml 10% DMSO/100 g b. w. were i.p. injected alone on the 11th day of the experiment (**Figure 1**). A previous study showed that 1–30% (v/v) DMSO solution have no effects on absence epileptic activity in WAG/Rij rats (Kovács et al., 2011a). To reveal the putative influence of A_1_R blockade on ketone supplements-evoked changes in SWD number, combined application of (i) i.p. saline injection (0.5 ml/100 g b. w.) with KSMCT gavage (2.5 g/kg/day, between 11th and 15th day of the experiment), (ii) i.p. 0.2 mg/kg DPCPX with KSMCT (2.5 g/kg/day) on the 6th day of KSMCT gavage (16th day of the experiment; DPCPX injection preceded the gavage by 30 min) and (iii) i.p. saline injection with KSMCT gavage (last day of the experiment; group 5) were carried out (**Figure 1**).

To investigate the effect of ketone supplements (KE and KSMCT) on blood glucose and βHB levels we measured them on the last (5th) control day (control), on the days of the 1st and the 7th ketone supplement gavage and on PT day (on the 10th, 11th, 17th, and 18th day of experiments, respectively; group 1 and group 2) (**Figure 1**). The body weight of rats were also measured before treatments by ketone supplement started (5th control day: control) and after the last (7th) ketone supplement treatments (on the 10th and 17th day of experiments, respectively; group 1 and group 2).

As changes in sleep-waking ratios may modulate SWD number in WAG/Rij rats (Coenen et al., 1991; Coenen and Van Luijtelaar, 2003) and as we had no prior data on putative effects of ketone supplements, DPCPX and combined application of DPCPX with KSMCT on sleep-waking stages, we investigated their effect (after 6th gavage of 2.5 g/kg KSMCT alone/group 2, i.p. 0.2 mg/kg DPCPX alone/group 3 and combined application of i.p. 0.2 mg/kg DPCPX with 2.5 g/kg KSMCT/group 5) not only on SWD number, SWD time (average time of SWDs) and discharge frequency within SWDs, but also on sleep-waking stages between 30 and 90 min. The effect of 2.5 g/kg KE (group 1) on SWD time and discharge frequency within SWDs was also investigated between 30 and 90 min. Evaluation of sleep-waking stages was performed offline by visual evaluation of the raw EEG. We distinguished wakefulness (predominantly beta/20–40 Hz and theta/6–8 Hz activity: wake; passive/active wake: without/with high slow waves of motor artifacts), SWS (sleep spindles/10–16 Hz, theta waves and some slow waves/2–4 Hz: light SWS; disappearance of sleep spindles and increasing ratio of high slow delta waves/0.5–4 Hz: deep SWS) and REM sleep (continuous theta activity without any motor artifacts) in 60 min epochs (Kovács et al., 2006).

All results (SWD number, SWD time, discharge frequency within SWDs, sleep-waking stages, b. w. as well as blood level of glucose and βHB) were expressed as means ± standard error of the mean. The pre-treatment control values were the grand average calculated (i) from the results of 5 control days (5-day control period; in relation to SWD number, SWD time, discharge frequency within SWDs and sleep-waking stages) or (ii) from the values measured on the last (5th) control days (in case of b. w. and blood level of glucose and βHB). Results were evaluated by One- or Two-way Repeated Measure Analysis of Variance and significance levels were determined by Dunnett’s Multiple Comparison Test and Bonferroni’s *Post hoc* Tests as were described previously (Kovács et al., 2015).

## Results

### Effect of Exogenous Ketone Supplements on SWD Number, Blood βHB, Glucose Levels, and Body Weight

**FIGURE 3:**
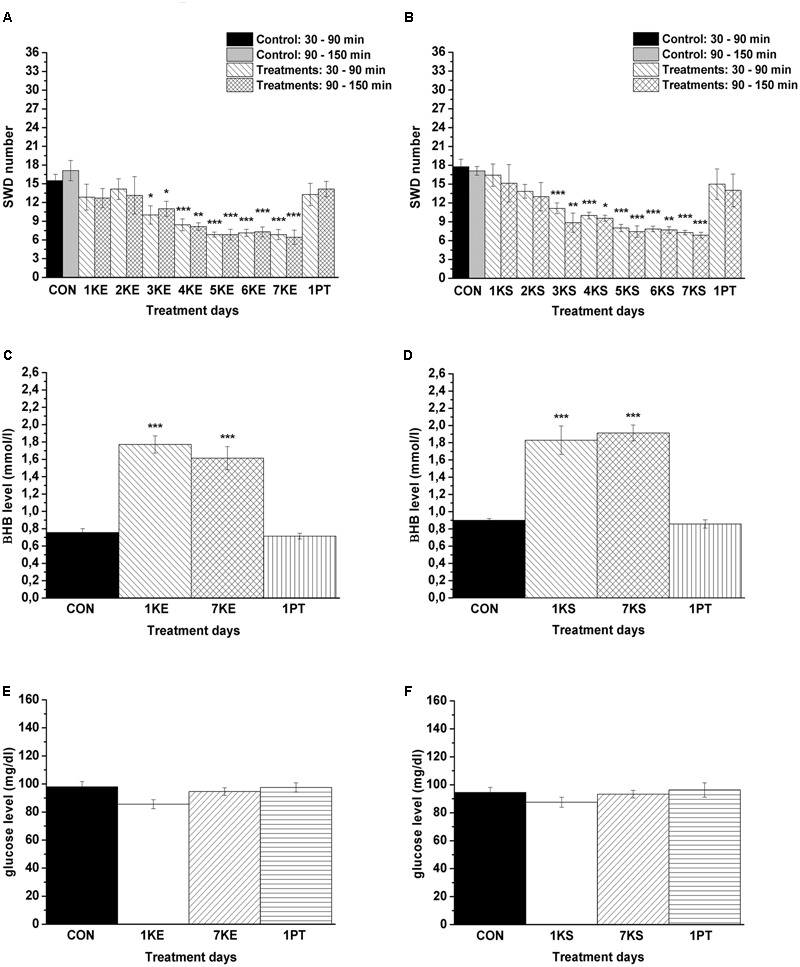
Effect of KE (gavage, 2.5 g/kg/day, group 1) and KSMCT (gavage, 2.5 g/kg/day, group 2) on SWD number (**A,B**, respectively) as well as on βHB and glucose levels (KE: **C,E**; KSMCT: **D,F**). 1KE, 1st KE application (gavage); 2KE, 2nd KE application and so on; 1KS, 1st KSMCT application (gavage); 2KS, 2nd KSMCT application and so on (KSMCT, ketone salt/KS + medium chain triglyceride/MCT); 1PT, post-treatment control experiment (2.5 g/kg water gavage); βHB, beta-hydroxybutyrate; CON, control; KE, ketone ester (1,3-butanediol – acetoacetate diester); SWD, spike-wave discharge. ^∗^*p* < 0.05, ^∗∗^*p* < 0.01, and ^∗∗∗^*p* < 0.001 level of significance.

Significant decrease in SWD number was demonstrated after both KE (2.5 g/kg/day, group 1) and KSMCT (2.5 g/kg/day, group 2) treatment between 3rd and 7th days of gavage from 30 to 150 min (**Figures 3A,B** and **Table 1**) compared to control levels. Moreover, SWD numbers were similar to the control levels on PT days (2.5 g/kg water gavage) (**Figures 3A,B** and **Table 1**).

**Table 1:** Effects of KE (gavage, 2.5 g/kg/day, group 1) and KSMCT (gavage, 2.5 g/kg/day, group 2) on SWD number.

Treatments	The effect of treatment	SWD number (mean ± SEM)	
	(one-way RM ANOVA;	and Dunnett’s Multiple Comparison Test	
	significance/*F*-value)	(significance/*q*-value)	
		30–90	90–150
**2.5 g/kg/day KE (**Figure 3A**, group 1)**			

**Control (CON)**	30–90 min: ^∗∗∗^/	15.5 ± 0.9	17.1 ± 1.6
1st KE treatment (1KE)	8.541	12.9 ± 2.1 -/1.602	12.7 ± 1.5 -/2.018
2nd KE treatment (2KE)	90–150 min: ^∗∗∗^/ 6.010	14.1 ± 1.7 -/0.827	13.1 ± 2.9 -/1.821
3rd KE treatment (3KE)		10.0 ± 1.5 ^∗^/3.324	11.1 ± 1.2 ^∗^/2.804
4th KE treatment (4KE)		8.4 ± 0.9 ^∗∗∗^/4.271	8.1 ± 0.6 ^∗∗^/4.115
5th KE treatment (5KE)		6.9 ± 0.4 ^∗∗∗^/5.218	6.9 ± 0.9 ^∗∗∗^/4.704
6th KE treatment (6KE)		7.1 ± 0.5 ^∗∗∗^/5.046	7.3 ± 0.8 ^∗∗∗^/4.508
7th KE treatment (7KE)		6.9 ± 0.8 ^∗∗∗^/5.218	6.4 ± 1.2 ^∗∗∗^/4.901
Post-treatment control experiment (1PT)		13.3 ± 1.8 -/1.343	14.1 ± 1.2 -/1.363

**2.5 g/kg/day KSMCT (**Figure 3B**, group 2)**			

**Control (CON)**	30–90 min: ^∗∗∗^/	17.8 ± 1.2	17.1 ± 0.7
1st KSMCT treatment (1KS)	14.000	16.4 ± 1.8 -/0.884	15.1 ± 2.9 -/0.855
2nd KSMCT treatment (2KS)	90–150 min: ^∗∗∗^/ 5.404	13.9 ± 1.1 -/2.575	13.0 ± 2.3 -/1.785
3rd KSMCT treatment (3KS)		11.1 ± 0.9 ^∗∗∗^/4.361	8.9 ± 1.5 ^∗∗^/3.582
4th KSMCT treatment (4KS)		10.0 ± 0.5 ^∗∗∗^/5.113	9.6 ± 0.5 ^∗^/3.273
5th KSMCT treatment (5KS)		8.0 ± 0.6 ^∗∗∗^/6.429	7.4 ± 0.9 ^∗∗∗^/4.202
6th KSMCT treatment (6KS)		7.9 ± 0.5 ^∗∗∗^/6.523	7.7 ± 0.5 ^∗∗^/4.078
7th KSMCT treatment (7KS)		7.3 ± 0.4 ^∗∗∗^/6.899	6.9 ± 0.5 ^∗∗∗^/4.450
Post-treatment control experiment (1PT)		15.0 ± 2.4 -/1.823	14.0 ± 2.6 -/1.351

*KE, ketone ester (1,3-butanediol – acetoacetate diester); KSMCT, ketone salt/KS + medium chain triglyceride/MCT; SWD, spike-wave discharge. ^∗^*p* < 0.05, ^∗∗^*p* < 0.01, and ^∗∗∗^*p* < 0.001 level of significance.*

Blood βHB and glucose levels were significantly increased and unchanged, respectively, after the 1st and 7th KE (2.5 g/kg/day, group 1) or KSMCT (2.5 g/kg/day, group 2) gavage (**Figures 3C–F** and **Table 2**) compared to control levels. The βHB and glucose levels returned to the baseline (control) levels on the PT day (**Figures 3C–F** and **Table 2**).

**Table 2:** Influence of KE (gavage, 2.5 g/kg/day, group 1) and KSMCT (gavage, 2.5 g/kg/day, group 2) on blood βHB and glucose levels.

Treatments	The effect of treatment	Blood βHB and glucose levels	
	(one-way RM ANOVA;	(mean ± SEM) and Dunnett’s Multiple Comparison Test	
	significance/*F*-value)	(significance/*q*-value)	
		βHB (mmol/l)	Glucose (mg/dl)
**2.5 g/kg/day KE (**Figures 3C,E**, group 1)**			

**Control (CON)**	Glucose: -/2.768	0.8 ± 0.1	98.0 ± 3.8
1st KE treatment (1KE)	βHB: ^∗∗∗^/50.090	1.8 ± 0.1 ^∗∗∗^/9.120	86.4 ± 3.7 -/2.538
7th KE treatment (7KE)		1.6 ± 0.1 ^∗∗∗^/7.707	94.6 ± 2.7 -/0.752
Post-treatment control experiment (1PT)		0.7 ± 0.1 -/0.385	97.6 ± 3.3 -/0.094

**2.5 g/kg/day KSMCT (**Figures 3D,F**, group 2)**			

**Control (CON)**	Glucose: -/1.552	0.9 ± 0.0	94.6 ± 3.7
1st KSMCT treatment (1KS)	βHB: ^∗∗∗^/41.180	1.8 ± 0.2 ^∗∗∗^/7.334	87.8 ± 3.6 -/1.633
7th KSMCT treatment (7KS)		1.9 ± 0.1 ^∗∗∗^/8.011	93.3 ± 2.7 -/0.299
Post-treatment control experiment (1PT)		0.9 ± 0.1 -/0.339	96.3 ± 5.2 -/0.399

*βHB, beta-hydroxybutyrate; KE, ketone ester (1,3-butanediol – acetoacetate diester); KSMCT, ketone salt/KS + medium chain triglyceride/MCT. ^∗∗∗^*p* < 0.001 level of significance.*

We did not find significant changes in b. w. of animals after KE (group 1) and KSMCT (group 2) application compared to control levels [b. w. of rats (g) on the 5th control day/after the 7th KE or KSMCT treatments ± SEM; control/KE: 323.3 ± 7.9/324.2 ± 8.1, *q* = 1.005; control/KSMCT: 335.6 ± 7.4/334.7 ± 6.7, *q* = 0.633].

### Effect of DCPX Alone and in Combination with KSMCT on SWD Number

**FIGURE 4:**
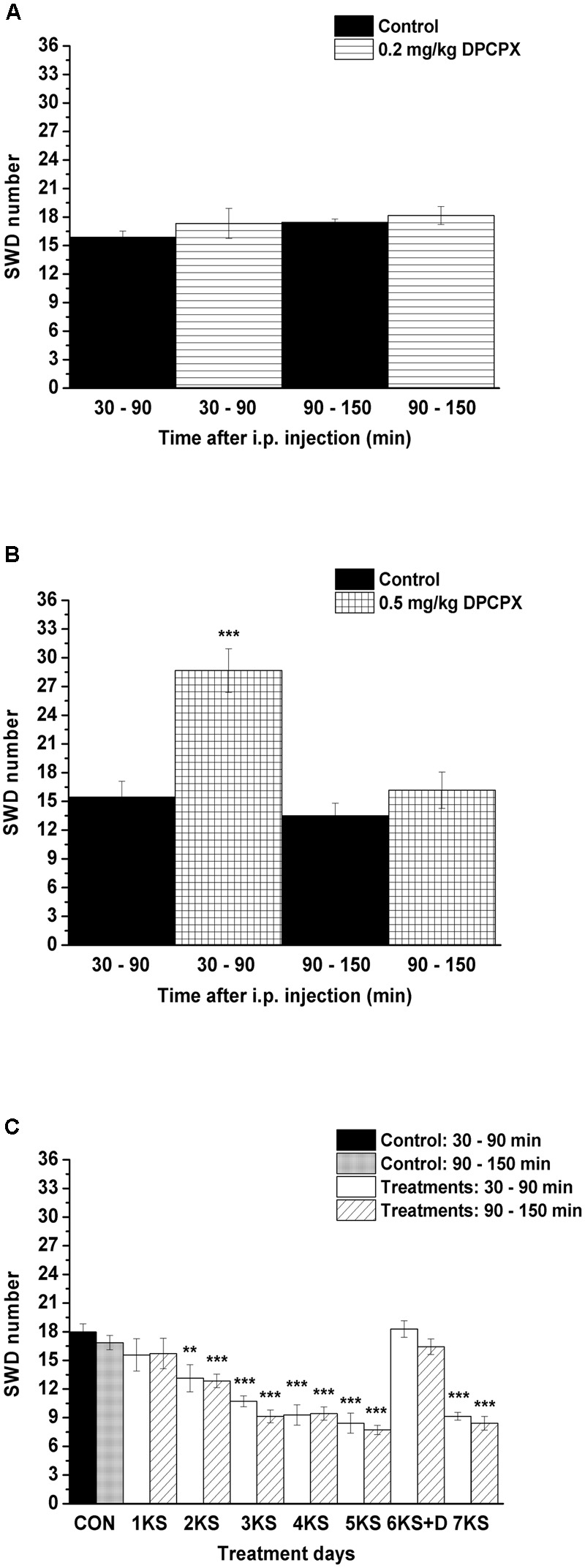
Effect of i.p. 0.2 mg/kg DPCPX (**A**, group 3), i.p. 0.5 mg/kg DPCPX (**B**, group 4) as well as KSMCT gavage (1KS – 5KS and 7KS) and combined application of i.p. 0.2 mg/kg DPCPX with KSMCT on day of 6th KSMCT gavage (6KS+D; **C**, group 5) on SWD number. 1KS, 1st KSMCT application (gavage); 2KS, 2nd KSMCT application and so on; 6KS+D, combined application of i.p. 0.2 mg/kg DPCPX with KSMCT on day of 6th KSMCT gavage (the gavage of KSMCT was preceded by i.p. injection of DPCPX by 30 min); CON, control; DPCPX, 1,3-dipropyl-8-cyclopentylxanthine; SWD, spike-wave discharge. ^∗∗^*p* < 0.01 and ^∗∗∗^*p* < 0.001 level of significance.

We demonstrated that a lower dose of DPCPX (i.p. 0.2 mg/kg, group 3) alone did not change the SWD number whereas the higher dose (i.p. 0.5 mg/kg, group 4) alone significantly increased the SWD number between 30 and 90 min after injection (**Figures 4A,B** and **Table 3**). However, the tendency in SWD number after the i.p. injection of higher DPCPX dose (control/DPCPX ± SEM: 13.5 ± 1.3/16.2 ± 1.9, *t* = 2.406) between 90 and 150 min was not significant (**Figure 4B** and **Table 3**).

**Table 3:** Changes in SWD number after i.p. injection of two doses of DPCPX (i.p. 0.2 mg/kg DPCPX: group 3; i.p. 0.5 mg/kg DPCPX: group 4).

Treatments	The effect of treatment	SWD number (mean ± SEM)	
	(two-way RM ANOVA;	and Bonferroni’s *post hoc* test	
	significance/*F*-value)	(significance/*t*-value)	
		30–90	90–150
**i.p. 0.2 mg/kg DPCPX (**Figure 4A**, group 3)**			

**Control**	-/1.979	15.9 ± 0.6	17.4 ± 0.4
DPCPX		17.3 ± 1.6 -/1.271	18.2 ± 0.9 -/0.719

**i.p. 0.5 mg/kg DPCPX (**Figure 4B**, group 4)**			

**Control**	^∗∗∗^/102.700	15.4 ± 1.7	13.5 ± 1.3
DPCPX		28.7 ± 2.3 ^∗∗∗^/11.930	16.2 ± 1.9 -/2.406

*DPCPX, 1,3-dipropyl-8-cyclopentylxanthine; SWD, spike-wave discharge. ^∗∗∗^*p* < 0.001 level of significance.*

A similar decrease in SWD number was observed after both KE and KSMCT gavage between 1st and 7th gavage days, but the change in SWD number was slightly smoother in relation to KSMCT (**Figures 3A,B**). Moreover, based on previous studies (Yudkoff et al., 2007; Masino et al., 2011; Rho, 2017), we hypothesized that the mechanism(s) of action on epileptic activity may be similar for KE and KSMCT. Thus, we used only KSMCT (2.5 g/kg/day) for combined application with DPCPX (group 5). In addition, we selected the lower dose of DPCPX (0.2 mg/kg) for combined application to decrease its putative side effects and because we wanted to inhibit (antagonize) A_1_Rs without increasing seizure activity (**Figures 4A,B**). As we did not find an effect of a lower DPCPX dose on behavior and as we demonstrated only a very slight and insignificant increase or decrease in SWD number, average SWD duration and discharge frequency within SWDs after its i.p. application alone (**Figure 4A**), we used this non-pro-epileptic dose of DPCPX (i.p. 0.2 mg/kg) in combination with KSMCT (2.5 g/kg/day) on the 6th day of KSMCT gavage, when, without DPCPX, KSMCT alone significantly decreased the SWD number (**Figure 3B**). As **Figure 4C** shows, i.p. 0.2 mg/kg DPCPX abolished the KSMCT-evoked decrease in SWD number on the 6th day of KSMCT gavage (**Figures 3B, 4C** and **Table 4**; group 5).

**Table 4:** Effect of KSMCT gavage (2.5 g/kg/day; between 1st and 5th KSMCT treatment/1KS – 5KS and on the 7th day of KSMCT application/7KS) and combined application of DPCPX (i.p. 0.2 mg/kg) with KSMCT gavage (DPCPX + 6th KSMCT treatment/6KS + D, group 5) on SWD number.

Treatments	The effect of treatment	SWD number (mean ± SEM)	
	(one-way RM ANOVA;	and Dunnett’s Multiple Comparison Test	
	significance/*F*-value)	(significance/*q*-value)	
		30–90	90–150
**i.p. 0.2 mg/kg DPCPX + 2.5 g/kg/day KSMCT (**Figure 4C**, group 5)**			

**Control (CON)**	30–90 min: ^∗∗∗^/	17.9 ± 0.9	16.9 ± 0.8
1st KSMCT treatment (1KS)	20.470	15.6 ± 1.7 -/2.400	15.7 ± 1.6 -/1.195
2nd KSMCT treatment (2KS)	90–150 min: ^∗∗∗^/ 32.340	13.1 ± 1.4 ^∗∗^/4.829	12.9 ± 0.7 ^∗∗∗^/4.181
3rd KSMCT treatment (3KS)		10.7 ± 0.6 ^∗∗∗^/7.257	9.1 ± 0.7 ^∗∗∗^/8.064
4th KSMCT treatment (4KS)		9.3 ± 1.1 ^∗∗∗^/8.686	9.4 ± 0.7 ^∗∗∗^/7.765
5th KSMCT treatment (5KS)		8.4 ± 1.0 ^∗∗∗^/9.543	7.7 ± 0.5 ^∗∗∗^/9.557
DPCPX + 6th KSMCT treatment (6KS+D)		18.3 ± 0.9 -/-0.314	16.4 ± 0.8 -/0.448
7th KSMCT treatment (7KS)		9.1 ± 0.4 ^∗∗∗^/8.829	8.4 ± 0.7 ^∗∗∗^/8.810

*DPCPX, 1,3-dipropyl-8-cyclopentylxanthine; KSMCT, ketone salt/KS + medium chain triglyceride/MCT; SWD, spike-wave discharge. ^∗∗^*p* < 0.01 and ^∗∗∗^*p* < 0.001 level of significance.*

### Effect of KE, KSMCT, DPCPX and Combined Application of DPCPX with KSMCT on Average SWD Duration, Discharge Frequency within SWDs and on Sleep Waking Stages

We did not find changes in the average SWD duration and discharge frequency within SWDs after application of KE (2.5 g/kg/day, 6th gavage, group 1), KSMCT (2.5 g/kg/day, 6th gavage, group 2), DPCPX (i.p. 0.2 mg/kg, group 3) alone and combined application of DPCPX (i.p. 0.2 mg/kg) with KSMCT (2.5 g/kg/day, 6th gavage, group 5) between 30 and 90 min (**Table 5**). Consequently, changes in the total time of SWDs paralleled the change in SWD number as shown in **Table 6** (last row) (**Figures 3B, 4A,C**).

**Table 5:** Influence of KE gavage (2.5 g/kg/day, 6th treatment, group 1), KSMCT gavage (2.5 g/kg/day, 6th treatment, group 2) and i.p. DPCPX (0.2 mg/kg, group 3) alone and combined application of i.p. DPCPX with KSMCT gavage (i.p. 0.2 mg/kg DPCPX + 6th KSMCT treatment, group 5) on average SWD duration and discharge frequency within SWDs between 30 and 90 min.

Treatments	Average SWD duration (sec; (mean ± SEM) and Bonferroni’s *post hoc* test (significance/*t*-value)	Discharge frequency within SWDs (Hz; mean ± SEM) and Bonferroni’s *post hoc* test (significance/*t*-value)
**2.5 g/kg/day KE (group 1)**		

**Control**	6.5 ± 0.6	7.9 ± 0.1
6th KE treatment	6.3 ± 0.7 -/0.913	7.8 ± 0.1 -/0.197

**2.5 g/kg/day KSMCT (group 2)**		

**Control**	6.6 ± 0.5	7.8 ± 0.1
6th KSMCT treatment	6.8 ± 0.7 -/0.652	7.9 ± 0.1 -/0.192

**0.2 mg/kg DPCPX (group 3)**		

**Control**	6.5 ± 0.8	7.8 ± 0.1
DPCPX	6.3 ± 0.8 -/0.044	7.8 ± 0.1 -/0.199

**i.p. 0.2 mg/kg DPCPX + 2.5 g/kg/day KSMCT (group 5)**		

**Control**	6.7 ± 0.7	7.8 ± 0.1
DPCPX + 6th KSMCT treatment	6.6 ± 0.7 -/0.522	7.8 ± 0.1 -/0.118

*DPCPX, 1,3-dipropyl-8-cyclopentylxanthine; KE, ketone ester (1,3-butanediol – acetoacetate diester); KSMCT, ketone salt/KS + medium chain triglyceride/MCT; SWD, spike-wave discharge.*

**Table 6:** Changes in total time of sleep-waking stages and SWDs after KSMCT gavage (2.5 g/kg/day, 6th treatment, group 2) and DPCPX injection (i.p. 0.2 mg/kg, group 3) alone and combined application of i.p. DPCPX with KSMCT gavage (i.p. 0.2 mg/kg DPCPX + 6th KSMCT treatment, group 5) between 30 and 90 min.

Sleep-waking stages and SWD	Total duration of sleep-waking stages and SWDs (sec; mean ± SEM) and Bonferroni’s *post hoc* test (significance/*t*-value)					
	2.5 g/kg/day KSMCT (6th KSMCT treatment, group 2)		i.p. 0.2 mg/kg DPCPX (group 3)		i.p. 0.2 mg/kg DPCPX + 2.5 g/kg/day KSMCT (6th KSMCT treatment, group 5)	
	Control	KSMCT	Control	DPCPX	Control	DPCPX + KSMCT
**Active wake**	702.7 ± 16.1	720.8 ± 15.4 -/1.027	675.8 ± 22.0	671.9 ± 19.3 -/0.141	688.2 ± 15.4	697.5 ± 16.9 -/0.440
**Passive wake**	704.1 ± 7.3	714.5 ± 10.4 -/0.598	731.9 ± 15.8	741.2 ± 26.6 -/0.346	704.7 ± 9.1	695.2 ± 16.8 -/0.447
**Light SWS**	990.2 ± 10.9	988.1 ± 18.1 -/0.121	974.2 ± 17.7	973.0 ± 23.8 -/0.042	981.0 ± 17.9	963.8 ± 11.6 -/0.817
**Deep SWS**	976.2 ± 15.9	1002.9 ± 17.2 -/1.525	1002.1 ± 24.6	997.3 ± 38.4 -/0.178	993.9 ± 20.3	1004.9 ± 23.2 -/0.517
**REM**	108.5 ± 3.2	115.3 ± 2.7 -/0.388	111.2 ± 7.5	106.5 ± 8.4 -/0.175	110.6 ± 4.0	112.0 ± 10.8 -/0.067
**SWD**	118.3 ± 7.3	58.4 ± 3.6 ^∗∗^/3.566	104.9 ± 3.9	110.0 ± 9.6 -/0.190	121.6 ± 5.9	126.6 ± 8.1 -/0.239

*DPCPX, 1,3-dipropyl-8-cyclopentylxanthine; KSMCT, ketone salt/KS + medium chain triglyceride/MCT; REM, rapid eye movement; SWD, spike-wave discharge; SWS, slow wave sleep; ^∗∗^*p* < 0.01 level of significance.*

The total time of sleep-waking stages was not changed after application of KSMCT (6th gavage, group 2), DPCPX (i.p. 0.2 mg/kg, group 3) alone and combined application of DPCPX with KSMCT (6th gavage, group 5) between 30 and 90 min (**Table 6**).

## Discussion

The current study is the first to demonstrate that the sub-chronic application of exogenous ketone supplements (both KE and KSMCT) by intragastric gavage decreased absence epileptic activity, and was abolished by pharmacologic inhibition of A_1_Rs in WAG/Rij rats.

It has been demonstrated that different types of ketogenic diets represent alternative treatment methods (metabolic-based therapies) for a broad range of seizure disorders, in which decreased epileptic activity may be related to elevations in serum ketone bodies, reduced glucose levels, suppression of insulin signaling as well as different neurotransmitter systems, such as GABAergic and adenosinergic system in different types of epilepsies (Ross et al., 1985; Van Delft et al., 2010; Sharma et al., 2015; Rho, 2017). Although the relationship between KD-evoked ketosis and seizure arrest is still unclear, it is widely accepted that high level ketosis has an important role in antiseizure/anticonvulsant effects of KD: βHB may alter metabolic pathways to increase both GABA and adenosine, which products have anticonvulsant and seizure suppressive effects (Gilbert et al., 2000; Bough et al., 2002; Neal et al., 2009; Van Delft et al., 2010; Sharma et al., 2015). Nevertheless, many patients have difficulty with compliance to the KD because of gastrointestinal symptoms including vomiting, diarrhea and abdominal pain (Ross et al., 1985; Veggiotti and De Giorgis, 2014). Therefore, testing new, non-pharmacological therapeutic strategies, such as application of exogenous ketone supplements instead of strict KD (Lee et al., 2016), which supplements may sustain therapeutic levels of ketosis are needed for some individuals. Exogenous ketone supplementation may generate corresponding beneficial therapeutic effect on epileptic seizures similar to the KD because (i) exogenous ketone supplementation-evoked influences may be similar to effects of KD (such as increased adenosine level), whose actions may mediate the effects of ketone supplementation on different types of epilepsies, (ii) among others, ketone bodies (e.g., βHB) may be responsible for the mediating effects of KD/ketosis as well as ketone supplementation on epileptic seizures due to altering Krebs cycle intermediates and GABAergic activity, and (iii) ketone supplementation (both KE and KSMCT) increase blood βHB levels and evoke nutritional ketosis at levels known to preserve brain homeostasis (**Figures 3C,D**) (Kwiterovich et al., 2003; Yudkoff et al., 2007; Masino et al., 2011; Poff et al., 2015; Ari et al., 2016; Kesl et al., 2016; Rho, 2017). Moreover, tolerability of oral/intragastric exogenous ketone supplementation in inducing nutritional ketosis is consistent with previous studies (Kesl et al., 2016), and provides a rationale for circumventing the dietary restriction of the KD or to enhance ketogenesis in less restrictive modified versions.

It was previously demonstrated that KD/ketosis may be effective against absence epileptic activity (Ross et al., 1985; Groomes et al., 2011; Thammongkol et al., 2012; Clemens et al., 2013; Kim et al., 2016; Lee et al., 2016). Our results suggest that not only KD, but also sub-chronically applied ketone supplements KE and KSMCT, administered by gavage, can increase βHB levels and decrease SWD number in freely moving WAG/Rij rats (**Figures 3A–D**). Moreover, SWD number and βHB level returned to the control level after both KE and KSMCT treatments (on the PT days). Thus, our results suggest that after ketone supplementation the absence epileptic activity can decrease in correlation with the increase in βHB level, which strengthens the support for nutritional ketosis as a means to suppress absence epileptic seizures (Ross et al., 1985; Groomes et al., 2011; Thammongkol et al., 2012; Clemens et al., 2013; Kim et al., 2016; Lee et al., 2016).

Moreover, it has been demonstrated that KD and ketone bodies (direct and/or indirect manner) among others may (i) increase adenosine signaling by both decreased expression/activity of adenosine kinase (which enzyme may metabolize adenosine) and enhanced βHB metabolism and, as a consequence, by A_1_Rs and (ii) enhance the inhibitory GABAergic effects in the brain by GABA_A_ receptors (Yudkoff et al., 2007; Masino et al., 2011, 2012; Sharma et al., 2015; Rho, 2017), which systems (GABAergic and adenosinergic) may have a role in the regulation of absence epileptic activity in WAG/Rij rats (Peeters et al., 1989; D’Alimonte et al., 2009; Kovács et al., 2015). It was demonstrated previously that activation of A_2A_Rs and GABA_A_ receptors may evoke increases in SWD number in WAG/Rij rats (D’Alimonte et al., 2009; Kovács et al., 2015; Lakatos et al., 2016), which results suggest that the modificatory effects of GABA_A_ receptors and A_2A_Rs in ketone supplementation-evoked decrease in absence epileptic activity can be excluded. Density of A_1_Rs is uneven in the central nervous system, expression/activity of A_1_Rs is decreased in the somatosensory cortex (focus) and thalamus in presymptomatic WAG/Rij rats, A_1_Rs has a role in the modulation of different types of epilepsies, and KDs may exert their effect on epileptic activity by means of A_1_Rs (D’Alimonte et al., 2009; Kovács et al., 2011b, 2014; Masino et al., 2011). Furthermore, (i) SWDs triggered by excessive hyperexcitability in the cortical focus of absence epilepsy genesis (Meeren et al., 2002; Coenen and Van Luijtelaar, 2003), (ii) brain areas, implicated in absence epilepsy genesis, such as somatosensory cortex, contain A_1_Rs (Cremer et al., 2009; Kovács et al., 2011b) and (iii) not only KD, but also ketone supplementation may modulate adenosinergic system by increased adenosine levels (Masino et al., 2011; Sharma et al., 2015; Rho, 2017). It has been demonstrated previously that inhibition of A_1_Rs by DPCX induces seizure-like bursting activity *in vitro* (Thümmler and Dunwiddie, 2000) and A_1_R knockout mouse exhibits electrographic seizures *in vivo* (Masino et al., 2011). Moreover, in accordance with cortical focus theory of absence epilepsy (Meeren et al., 2002; Coenen and Van Luijtelaar, 2003), activation of inhibitory A_1_Rs, which may decrease the excessive hyperexcitability in the somatosensory cortex (cortical focus) directly and indirectly by synaptic inhibition with reducing Ca^2+^ influx through voltage-dependent Ca^2+^ channels, and activation of both ATP-sensitive potassium (K_ATP_) channels and G protein-gated inwardly rectifying potassium channels hyperpolarizing neuronal membranes (Kovács et al., 2014; Lusardi et al., 2015; Rho, 2017), may decrease absence epileptic activity. In addition, ketosis/ketone bodies may indirectly activate K_ATP_ channels (Ma et al., 2007). All of these inhibitory effects may be implicated in the modulatory effect of A_1_Rs on KE and KSMCT treatment-evoked changes in epileptic seizures (**Figure 4C**), and/or strengthened the aggravating effect of higher dose of DPCPX on SWD number (**Figure 4B**) in WAG/Rij rats.

As to the antiepileptic effect of ketone supplementation, additional mechanisms of action similar to KD cannot be excluded (Rho, 2017). For example, depending on the diet formulation, KD/ketosis may result in (i) increased level of two potentially anti-convulsant fatty acids decanoic acid and octanoic acid, (ii) modified synaptic vesicle recycling by means of βHB (Chang et al., 2013; Hrynevich et al., 2016), (iii) appearance of adenosine-associated epigenetic mechanism(s) (Lusardi et al., 2015), (iv) increased threshold for calcium-induced mitochondrial permeability transition (Kim et al., 2015), and (v) decrease in extracellular glutamate release (Juge et al., 2010), which effects may also alleviate epileptic seizures. For example, it has been demonstrated that MCT may evoke anti-seizure effect by decanoic acid through AMPA receptor inhibition *in vitro* (Chang et al., 2016). Consequently, several mechanisms of action might be involved in KD- and ketone supplementation-evoked antiepileptic/antiseizure effects. However, our data are not sufficient to interpret all of the putative mechanisms, by which (i) exogenous ketone supplementation exerts its antiepileptic effects and (ii) A_1_Rs modulate effects of ketone supplements on seizure activity.

## Conclusion

The administration of exogenous ketone supplements without dietary restrictions (standard rodent diet) may be an effective alternative way to the KD not only to reach and maintain nutritional ketosis, but also to reduce epileptic activity, and which can be reversed by inhibition of A_1_Rs. Our results on WAG/Rij rats and modulation of ketone supplementation-evoked effects on absence epileptic activity provided an opportunity to investigate some new aspects of the pathophysiology of absence epilepsy, several signaling pathways, which are still poorly understood, influence of ketone supplementation on epileptic activity and the correlation between antiepileptic activity of ketone supplementation and their potential to induce ketosis. Our results may contribute to the development of effective antiepileptic strategies such as ketone supplementation not only against absence epilepsy (e.g., childhood absence epilepsy), but also for other therapy-resistant types of epilepsies, which may afford long-lasting seizure protection. Therefore, further studies are needed to reveal the exact molecular and neuropharmacological effects of exogenous ketone supplementation-induced decrease in absence epileptic activity and to disclose the putative link(s) between exogenous ketone supplements-evoked and adenosine-generated effects on seizure activity.

## Author Contributions

ZK: conception and design of experiments, data collection, interpretation of data, and writing manuscript, DD: interpretation of data, writing manuscript, AD: data analysis, writing manuscript, CA: conception and design of experiments, writing manuscript.

## Conflict of Interest Statement

InternationalPatent # PCT/US2014/031237, University of South Florida, D. P. D’Agostino, S. Kesl, P. Arnold, “Compositions and Methods for Producing Elevated and Sustained Ketosis.” The other authors declare that the research was conducted in the absence of any commercial or financial relationships that could be construed as a potential conflict of interest.

## Abbreviations

A_1_R: adenosine A_1_ receptor
A_2A_R: adenosine A_2A_ receptor
AcAc: acetoacetate
ATP: adenosine triphosphate
βHB: beta-hydroxybutyrate
DMSO: dimethyl sulfoxide
DPCPX: 1,3-dipropyl-8-cyclopentylxanthine
EEG: electroencephalogram
FFT: fast Fourier transform
GABA: gamma-aminobutyric acid
i.p.: intraperitoneal
K_ATP_ channels: ATP-sensitive potassium channels
KD: ketogenic diet
KS: ketone salt
KSMCT: ketone salt/KS + medium chain triglyceride/MCT
MCT: medium chain triglyceride
PT day: post-treatment control experiment/day
REM sleep: rapid eye movement sleep
S.E.M.: standard error of the mean
SWD: spike-wave discharge
SWS: slow wave sleep
WAG/Rij: Wistar Albino Glaxo/Rijswijk
